# 

**Published:** 1991-03

**Authors:** 


W1 WE403

V.106 NO.1 1991

C.01---------SEQ: SR0068788

TI: WEST OF ENGLAND MEDICAL

JOURNAL

### WEST OF ENGLAND MEDICAL JOURNAL

Volume 106(i) March 1991

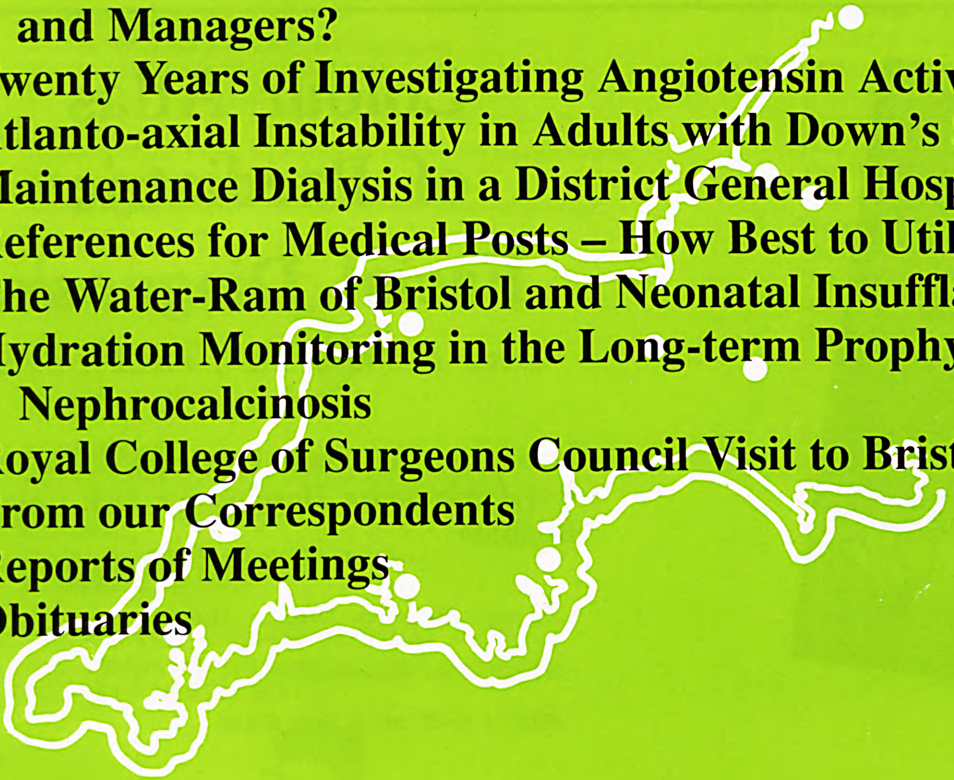

What Can We Do to Improve Relationships between Clinicians and Managers?
Twenty Years of Investigating Angiotensin Activity
Atlanto-axial Instability in Adults with Down's Syndrome
Maintenance Dialysis in a District General Hospital
References for Medical Posts – How Best to Utilise Them
The Water-Ram of Bristol and Neonatal Insufflation
Hydration Monitoring in the Long-term Prophylaxis of Nephrocalcinosis
Royal College of Surgeons Council Visit to Bristol
From our Correspondents
Reports of Meetings
Obituaries

NATIONAL LIBRARY
NOV 7 1991
OF MEDICINE

FORMERLY BRISTOL MEDICO-CHIRURGICAL JOURNAL

ISSN: 0960 6440 NLM Code B5Q

